# The role of kidney dysfunction in COVID-19 and the influence of age

**DOI:** 10.1038/s41598-022-12652-0

**Published:** 2022-05-23

**Authors:** Edoardo La Porta, Paola Baiardi, Lorenzo Fassina, Alessandro Faragli, Simone Perna, Federico Tovagliari, Ilaria Tallone, Giuseppina Talamo, Giovanni Secondo, Giovanni Mazzarello, Vittoria Esposito, Matteo Pasini, Francesca Lupo, Giacomo Deferrari, Matteo Bassetti, Ciro Esposito

**Affiliations:** 1 Division of Nephrology, Dialysis and Transplantation, Scientific Institute for Research and Health Care, IRCCS Istituto Giannina Gaslini, via Gerolamo Gaslini 5, 16147 Genoa, Italy; 2 Department of Internal Medicine (DiMi), University of Genoa, Genoa, Italy; 3 Scientific Direction, Istituti Clinici Scientifici Maugeri IRCCS, Pavia, Italy; 4 Department of Electrical, Computer and Biomedical Engineering, University of Pavia, Pavia, Italy; 5 Department of Cardiology, Charité-University Medicine Berlin, Campus Virchow Klinikum, Berlin, Germany; 6 Department of Biology, Sakhir Campus, College of Science, University of Bahrain, Sakhir, Bahrain; 7 General Medicine Department, Ospedale Evangelico, Genoa, Italy; 8 Nephrology Department, Ospedale San Paolo, Savona, Italy; 9 Emergency Department, Ospedale Evangelico, Genoa, Italy; 10 Infectious Disease Clinic Genoa University, Ospedale San Martino, Genoa, Italy; 11 Nephrology and Dialysis Unit, Istituti Clinici Scientifici Maugeri IRCCS, Pavia, Italy; 12 Department of Cardionephrology, Istituto Clinico Ligure Di Alta Specialità (ICLAS), GVM Care and Research, Rapallo, GE Italy; 13 University of Pavia, Pavia, Italy

**Keywords:** Diseases, Nephrology, Risk factors

## Abstract

COVID-19 is strongly influenced by age and comorbidities. Acute kidney injury (AKI) is a frequent finding in COVID-19 patients and seems to be associated to mortality and severity. On the other hand, the role of kidney dysfunction in COVID-19 is still debated. We performed a retrospective study in a cohort of 174 hospitalized COVID-19 patients in Italy from March 3rd to May 21st 2020, to investigate the role of kidney dysfunction on COVID-19 severity and mortality. Moreover, we examined in depth the relationship between kidney function, age, and progression of COVID-19, also using different equations to estimate the glomerular filtration rate (GFR). We performed logistic regressions, while a predictive analysis was made through a machine learning approach. AKI and death occurred respectively in 10.2% and 19.5%, in our population. The major risk factors for mortality in our cohort were age [adjusted HR, 6.2; 95% confidence interval (CI) 1.8–21.4] and AKI [3.36 (1.44–7.87)], while, in these relationships, GFR at baseline mitigated the role of age. The occurrence of AKI was influenced by baseline kidney function, D-dimer, procalcitonin and hypertension. Our predictive analysis for AKI and mortality reached an accuracy of ≥ 94% and ≥ 91%, respectively. Our study scales down the role of kidney function impairment on hospital admission , especially in elderly patients. BIS-1 formula demonstrated a worse performance to predict the outcomes in COVID-19 patients when compared with MDRD and CKD-EPI.

## Introduction

The coronavirus disease 2019 (COVID-19), caused by Severe acute respiratory syndrome coronavirus 2 (SARS-CoV-2), has rapidly spread all around the world after its outbreak in Whuan, Hubei, China in December 2019.

COVID-19 infection can quickly progress from severe to critical stage with the need for intensive care; in addition, the infection causes a high rate of ARDS (Acute Respiratory Distress Syndrome) often followed by death. In this clinical outlook, several studies have also reported the impact of age and comorbidity on the progression of COVID-19 infection^1^.

In several studies, kidney dysfunction has been associated with COVID-19 infection, severe and critical COVID-19, and mortality^2,3^. However, despite a variety of evidence from observational studies and metanalysis, the real impact of Acute Kidney Injury (AKI) as a clinical risk factor is still lacking^4^. The incidence of Chronic Kidney Disease (CKD) in COVID-19 hospitalized patients is also not clearly defined, and it is probably influenced by demographic and epidemiologic characteristics^5^. The role of CKD in COVID-19 severity is even more debated, probably as a result of the difficulty in differentiating CKD from AKI in patients hospitalized during the pandemic outbreak^6^. Studies and metanalysis showed an increased risk only for CKD stage 4–5, with a lack of evidence for the impact of the disease at the earlier stages. A recent study evidenced that kidney dysfunction on admission was a greater risk factor for younger than for elderly patients in COVID-19^6^. Moreover, CKD prevalence is higher among the elderly and patients with comorbidities such as diabetes, arterial hypertension and cardiovascular disease, which are also associated with severe COVID-19 and represent possible confounding factors.

Many variables are probably implicated in COVID-19 mortality and severity and, among them, kidney injury could be particularly relevant, also due to its growing prevalence^8^.

Moreover, the role of eGFR in the elderly is debated among nephrologists^9^, and different eGFR formulas have been developed specifically for elderly patients^10^.

The aim of this retrospective study is to evaluate the role of kidney dysfunction in the progression and severity of COVID-19 infection, by analyzing the relationship between age and kidney dysfunction on clinical outcomes, and evaluating the performance of specific eGFR equations to predict the outcomes in this setting.

## Materials and methods

### Study design

This was a retrospective study conducted at the *Ospedale Evangelico Internazionale* of Genova (Italy) approved by the Regional Ethic Committee on 02/07/2020 (N. CER Liguria registry number: 257/2020-ID 10476). All methods were performed in accordance with relevant guidelines/regulations and in accordance with the Declaration of Helsinki. Study population included 174 patients admitted to the hospital with Sars-Cov2 positive RT-PCR nasopharyngeal swab, from March 3rd, 2020 to May 21st, 2020. The hospital was totally converted to COVID-19 management during the pandemic outbreak. Multidisciplinary teams composed by internist, pneumologist, nephrologist, infectious disease specialist, emergency care physician and anesthetist were established. The hospital facility was divided in three different levels of care: mild disease, severe disease and critical care. All patients that underwent endotracheal intubation (ETI) were admitted to critical care unit.

For each patient, data were acquired from paper charts and electronic medical records and anonymized before analyses. Due to the emergency scenario, the high biological hazard, and the need for improving scientific knowledge on COVID-19, the Regional Ethic Committee (CER Liguria) approved this research without requirement of a written informed study-specific consent by the patients because of isolation precautions, and in consideration of the retrospective nature of the study.

### Inclusion and exclusion criteria

The analysis included all COVID-19 patients aged ≥ 18 years who resulted positive to at least 2 Sars-Cov2 positive RT-PCR nasopharyngeal swabs, and whose radiological findings documented pulmonary disease (via either X-ray or CT scan), and whose blood oxygen saturation was < 95% evidenced with arterial blood gas analysis.

The exclusion criteria were age < 18 years and death in the first 24 h after admission. Moreover, we excluded patients with less than 2 serum creatinine examinations and with unknown medical history. Of the 182 patients admitted to the hospital, 174 constituted the study group after the application of inclusion and exclusion criteria.

### Data collection

The variables collected at the time of hospital admission were age, gender, blood pressure, comorbidities including cardiovascular disease (CVD), diabetes, hypertension, chronic obstructive pulmonary disease (COPD), malignancies, medications (dexamethasone, oseltamivir, ritonavir/darunavir, hydroxychloroquine, heparin, tocilizumab, antihypertensive drugs), and biohumoral laboratory data: serum creatinine (sCr), blood urea nitrogen, ferritin, c-reactive protein (CRP), procalcitonin, lactate dehydrogenase (LDH), transaminase, blood cells count. CVD was defined as any of the following: previous episodes of myocardial infarction, revascularization procedures, strokes, acute heart failure, peripherical vascular disease or LVEF < 55%. Serum creatinine and blood urea nitrogen values were collected for 7 days after hospital admission. Glomerular filtration rate (GFR) was estimated based on sCr at the baseline.

### Definitions

- Baseline sCr was defined as the sCr value on admission;

- eGFR: was calculated using different equations: CKD EPI, MDRD, while in patients over 70 or 80 years the CKD BIS 1 was used^11^; Thus, 4 different variables were generated for the analysis: MDRD and CKD-EPI for the whole population, CKD-EPI for patients < 70 years and BIS-1 for patients ≥ 70 years (BIS-1 over 70), and CKD-EPI for patients < 80 years and BIS-1 for patients ≥ 80 years (BIS-1 over 80);

- AKI were defined and divided in different stages according to KDIGO criteria (KDIGO, Kidney Int Suppl.2, 2012, 1–138) i.e. AKI 1 increase in serum creatinine at least of 0.3 mg/dL within 48 h or at least 1.5–1.9 times baseline creatinine within 7 days; AKI 2 increase in serum creatinine 2.0–2.9 times baseline; AKI 3 increase serum creatinine 3.0 times baseline or increase serum creatinine ≥ 4.0 mg/dL or initiation of kidney replacement therapy. Urine criteria were not used in this analysis.

### Study endpoints

(a) Primary endpoints were AKI and mortality;

(b) Secondary endpoint was severity of the disease defined as the need for high oxygen fluxes (FiO_2_ ≥ 60%), continuous positive airway pressure (CPAP) or mechanical ventilation after endotracheal intubation (ETI,) and were evaluated as a composite endpoint.

### Statistical analysis

Patients’ characteristics are expressed as frequency (n) and percentage (%) for qualitative variables; quantitative variables are expressed as mean ± standard deviation, or median and Interquartile Range (IQR, i.e. difference between 75 and 25 percentiles) for non-normally distributed variables.

Univariate analysis was carried out for baseline characteristics of different AKI endpoints and for non-kidney short-term clinical outcomes. Continuous variables were compared by unpaired Student’s *t* test or Mann Whitney U test whenever required. Categorical variables were compared by χ^2^ test and odds ratios (OR) with 95% Confidence Intervals (95% CI) were reported. A two-sided *p* value < 0.05 was considered significant. Kaplan–Meyer curves were performed in order to model survival time and Logrank test was applied to assess comparisons between curves. Cox analysis with a stepwise approach was carried out to evaluate the effect of age (cut-off set at 70 years, being 70 years the median of the distribution) and AKI (yes/no) and other kidney dysfunction variables (creatinine and eGRF) on the survival. Crude and adjusted Hazard ratios (HR) and relative 95% CI were also estimated.

We were also interested in the prediction of some binary outcomes such as mortality (yes/no), composite endpoint (yes/no), and AKI (yes/no) via a well-known Machine Learning tool: using the above-listed variables or predictors, a tenfold cross-validation was applied to calculate the accuracy (%) of prediction by the MATLAB® Classification Learner application (method: Decision Tree via the built-in function “fitctree”; The MathWorks, Inc., Natick, MA). In detail, via the tenfold cross-validation, for 10 times, a 10% of the patients were not used to train but to blindly validate the predictive model; the cross-validation is a good practice to avoid the over-fitting which is an undesired memorization of the data reducing the predictive ability of the model. In addition, we assumed that the choice of only a single indicator of kidney functionality (serum creatinine, or the estimated CKD-EPI, BIS-1 over-70, BIS-1 over-80, MDRD) together with the remaining and above-listed variables could give a good performance in predicting the binary outcomes (mortality, composite endpoint, AKI).

## Results

### Characteristics of patients with COVID-19

Characteristics of the 174 patients are shown in Table 1. The mean age of study participants was 69.1 ± 15.7 years with a male preponderance (63.2%). The most common comorbidities were hypertension (48.9%), CVD (24.1%) diabetes (12.6%). GFR at baseline was 88.9 ± 33.4 with MDRD, 79.8 ± 23.9 with CKD-EPI, and 75.5 ± 25.8 mL/min with CKD-EPI and BIS-1 (for patients 70 years older). The median creatinine at baseline was 82.68 (IQR = 28.3 micromol/L). Patients with eGFR < 60 mL/min at baseline were 16.6% with MDRD, 19.5% with CKD-EPI, 27.6% with BIS-1 over 80 and, 28.7% over 70, respectively. The median duration of hospitalization was 19 (IQR = 21 days).

**Table 1:** Clinical characteristics of patients (whole sample and stratified by AKI).

	All patients (n = 174)		AKI				
			No (n = 156)		Yes (n = 18)		p*
	Mean (median)	SD (IQR)	Mean (median)	SD (IQR)	Mean (median)	SD (IQR)	
Age (years)	69.06	15.69	68.03	15.90	77.98	10.43	0.01
eGFR (MDRD)	88.90	33.42	91.07	31.54	70.79	43.16	0.015
eGFR (CKD-EPI;BIS-1 over-80 years)	76.71	25.77	79.29	24.87	55.50	23.50	< 0.001
eGFR (CKD-EPI; BIS-1 over-70 years)	75.48	25.77	77.97	24.99	55.0	23.50	< 0.001
eGFR (CKD-EPI)	79.78	23.88	82.10	22.92	60.72	23.67	< 0.001
Creatinine (mg/dL)	0.93 (0.88)	0.40 (0.32)	0.89 (0.85)	0.30 (0.31)	1.29 (1.09)	0.81 (0.40)	0.001
Creatinine (micromol/L)	82.68 (77.81)	35.50 (28.29)	78.96 (75.60)	26.27 (26.53)	113.67 (95.94)	72.05 (35.37)	0.002
Creatinine 48 h (mg/dL)	0.97 (0.83)	0.60 (0.37)	0.84 (0.80)	0.30 (0.27)	1.73 (1.60)	1.14 (0.76)	< 0.001
Creatinine 7 days (mg/dL)	0.97 (0.80)	0.57 (0.47)	0.82 (0.78)	0.29 (0.26)	1.89 (1.59)	0.87 (0.42)	< 0.001
Blood urea nitrogen (mg/dL)	40.23	22.92	38.70	22.04	52.40	26.77	0.029
Blood urea nitrogen 48 h (mg/dL)	42.85	27.91	37.49	18.97	73.60	46.79	0.010
White blood cells (n°/cc)	7014.77 (5590)	6826.20 (3740)	6939.2 (5445)	7041.9 (3260)	7661.7 (7550)	4681.7 (4970)	0.265
Neutrophiles (n°/cc)	4957.08 (3800)	3432.36 (3400)	4813.4 (3800)	3272.9 (2800)	6178.3 (5900)	4502.3 (4820)	0.168
Limphocytes (n°/cc)	1058.78	560.43	1078.6	567.9	888.9	472.2	0.175
Platelets (n°)	212.91	90.33	211.2	87.3	227.3	114.6	0.475
LDH (IU/L)	277.38 (244)	116.32 (128)	270.6 (244.5)	105.0 (129)	338.1 (242)	183.9 (178)	0.259
GOT (IU/L)	39.92 (31)	32.57 (24)	40.07 (30)	33.85 (26)	38.53 (35)	17.29 (15)	0.337
GPT (IU/L)	37.89 (27)	37.52 (28)	38.77 (27)	38.97 (29)	30.06 (26.5)	19.60 (23.5)	0.576
CRP (mg/L)	69.96 (47.0)	70.83 (98.55)	68.57 (47.0)	68.83 (96.6)	81.92 (38.5)	87.49 (109.2)	0.774
Procalcitonin (ng/mL)	0.90 (0.10)	4.00 (0.16)	0.57 (0.10)	2.39 (0.17)	3.36 (0.20)	9.45 (0.50)	0.008
Ferritin (ng/mL)	832.46 (573)	907.65 (723)	758.3 (499)	831.5 (689)	1329.5 (844)	1253.7 (1686)	0.097
D-dimer (ng/mL)	2548.91 (948)	5157.41 (1764)	2269.2 (746)	5173.2 (1446)	4478.8 (2094)	4856.7 (4535)	0.002
Systolic blood pressure (mmHg)	127.02	20.37	128.00	20.01	121.18	22.12	0.203
Diastolic blood pressure (mmHg)	73.60	12.35	74.31	11.89	69.41	14.46	0.131
Lenght of hospitalization (days)	25.20 (19)	20.36 (21)	24.94 (18.5)	20.09 (20)	27.44 (19)	23.08 (21)	0.683
Time between the onset of symptoms and discharge/death (days)	25.47	16.12	25.92	16.37	21.63	13.61	0.315

Median and Interquartile Range are shown for non normally distributed variables only.

*Comparison between AKI and no AKI: Student’s t test or Mann–Whitney test, as appropriate.

AKI = Acute Kidney Injury; SD = Standard Deviation; IQR = Interquartile Range; eGFR = Estimated Glomerular Filtration Rate; MDRD = Modification of Diet in Renal Disease Study; CKD-EPI = *Chronic Kidney Disease* Epidemiology Collaboration; BIS-1 = Berlin Initiative Study 1 Equation; LDH = *Lactate Dehydrogenase; GOT* = Glutamic Oxaloacetic Transaminase; GPT = Glutamate Pyruvate Transaminase; CRP = C-Reactive Protein.

### Variables associated with AKI in COVID-19 patients

The occurrence of AKI in our population was 10.2%. Compared to patients that did not develop AKI, patients with AKI showed significantly higher values of creatinine, and blood urea nitrogen values at the baseline: 113.67 ± 72.05 vs. 78.96 ± 26.27 micromol/L, and 52 ± 27 vs. 39 ± 22 mg/dL, respectively. Biochemical variables correlated to AKI were: procalcitonin (3.36 ± 9.45 vs. 0.57 ± 2.39 ng/mL, *p* = 0.008), and D-dimer (4479 ± 4857 vs. 2269 ± 5173 ng/mL, *p* = 0.002). Hypertension represents a risk factor for AKI (OR 6.14; 95% CI 1.71–22.1), but not diabetes or CVD.

(Tables 1, Supplementary materials 1).

### Variables associated with mortality in COVID-19 patients

The overall mortality was 19.5%. The average time between disease onset and death was 19 ± 12.8 days, whereas median time before discharge was 27.1 ± 16.6 days. Compared to survivors, non-survivors were older (80.2 ± 7.8 vs. 66.5 ± 16.0 years *p* < 0.001), presented higher baseline values of creatinine (1.14 ± 0.41 vs. 0.9 ± 0.39 mg/dL or 100.02 ± 35.51 vs. 78.79 ± 34.55 micromol/L *p* = 0.002), and blood urea nitrogen (59.78 ± 30.7 vs. 35.47 ± 17.61 mg/dL). Moreover, in non-survivors, neutrophils, GOT, PCR, procalcitonin were higher, while lymphocytes and diastolic blood pressure were lower (Supplementary material Table 2). Creatinine and blood urea nitrogen were also significantly higher between 48 h and 7 days, in the non-survivors group. No differences were found for gender and LDH values.

Patients with AKI presented higher percentage of death compared with non-AKI patients (50% vs. 16%) and an increase risk for mortality (OR 5.16 95% CI 1.86–14.3). We observed higher percentages of deaths (71.4% vs. 36.4%) in moderate-severe AKI (stages 2–3) compared to mild AKI (stage 1) and an increase in risk for mortality from mild AKI (2.2; IC95%: 0.8–6.6) to moderate-severe AKI (8.4; IC95%: 3.1–22.7). Univariate analyses identified the following additional risk factors for mortality: arterial hypertension (OR 3.12; 95% CI 1.39–7.02), diabetes (OR 3.46 95% CI 1.34–8.97), COPD (OR 3.45; 95% CI 1.20–9.89), CVD (OR 4.52; 95% CI 2.03–10.06).

The use of antiviral drugs was also associated with increased risk of mortality, oseltamivir (OR 3.62; 95% CI 1.61–8.16), ritonavir/darunavir (OR 3.40; 95% CI 1.48–7.83), while dexamethasone seemed to be protective with an association with the outcome close to statistical significance (OR 0.45; 95% IC 0.19–1.07) and no differences were found for hydroxychloroquine, heparin, and tocilizumab (Supplementary materials Table 3).

**Figure 1:**
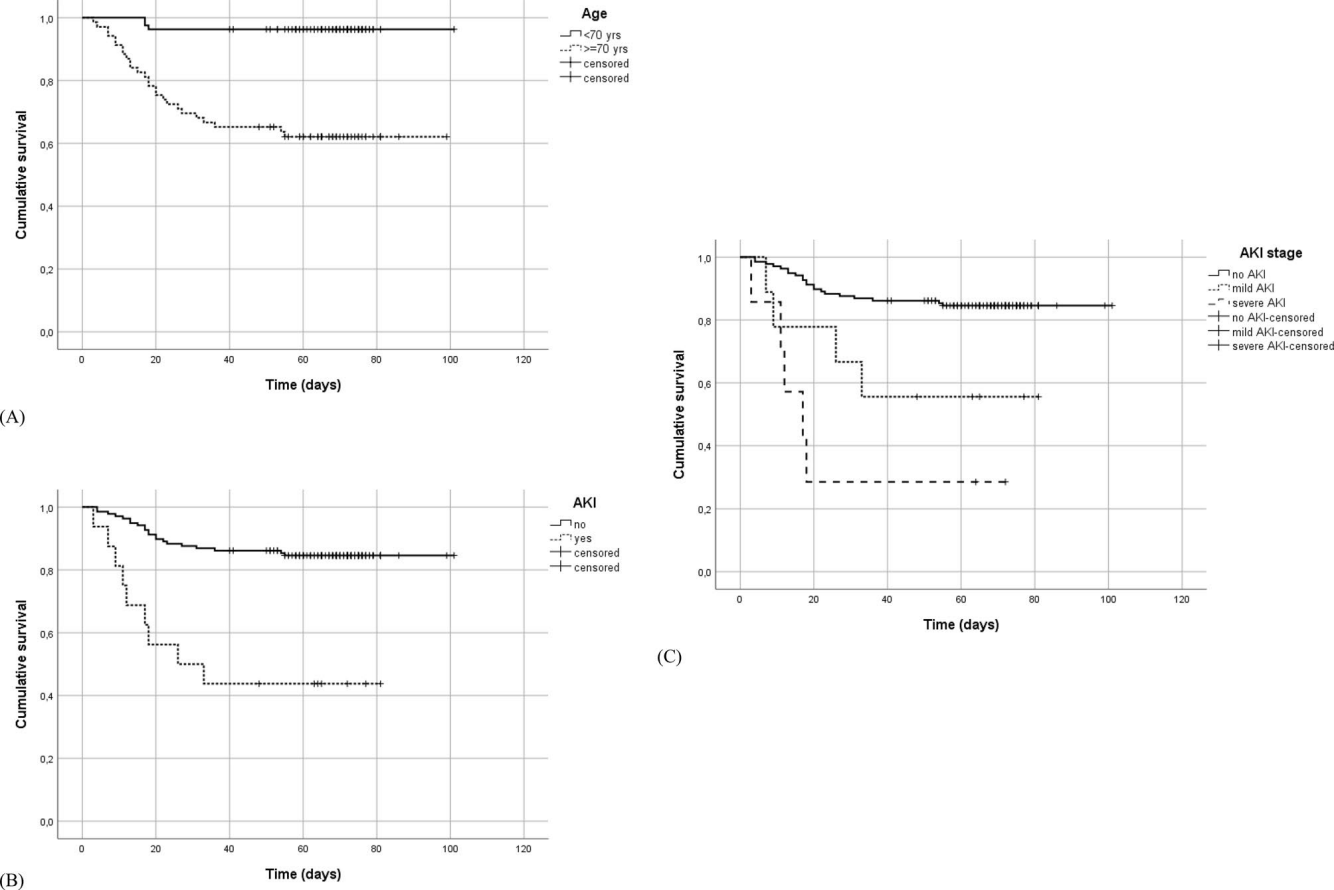
Kaplan–Meier plots of mortality. Curves represent mortality in (**A**) = patients over and under 70 years old—Logrank test: *p* < 0.001. (**B**) = patients with and without occurrence of Acute Kidney Injury (AKI)—Logrank test: *p* < 0.001. (**C**) = patients without and with mild (stage 1) and severe (stage 2–3) Acute Kidney Injury (AKI)—Logrank test: *p* < 0.001, pairwise comparisons: no AKI vs. mild AKI: *p* = 0.014; no AKI vs. severe AKI: *p* < 0.001; mild AKI vs. severe AKI: *p* = 0.227.

Survival analysis identified two major predictors for mortality, age and AKI. Kaplan–Meier plots (Fig. 1) pointed out significantly shorter onset of mortality for the older old and the AKI groups in comparison to the younger population and no-AKI (Logrank test: *p* < 0.001). Moreover, severe AKI showed significantly shorter onset of mortality compared with mild AKI. Among other kidney dysfunction variables, eGFR showed an effect on mortality by mitigating the role of age but not that of AKI (see Table 2).

**Table 2:** Crude and adjusted Hazard Ratios (HR) for mortality.

Risk Factor	HR (95% CI)	HR_adj_ (95% CI)*
Age ≥ 70 years *(ref* < *70 years)*	10.73 (3.22 – 35.7)	6.2 (1.80 – 21.4)
AKI *(ref no AKI)*	3.79 (1.71 – 8.38)	3.36 (1.44 – 7.87)
Mild AKI *(ref no AKI)*	2.24 (0.8 – 6.6)	1.90 (0.62 – 5.82)
Moderate-Severe AKI *(ref no AKI)*	8.41 (3.1 – 22.7)	8.43 (2.96 – 24.02)

*Adjusted by creatinine (mg/dL) and eGFR (MDRD).

### Variables associated with COVID-19 severity

When considering the need for CPAP, ETI, FiO_2_ ≥ 60% or mortality, 44.8% of the patients presented at least one of the mentioned conditions (composite endpoint).

Variables significantly associated with the composite endpoint were: sCr at baseline (1.05 ± 0.52 vs. 0.85 ± 0.25 mg/dL *p* = 0.002), blood urea nitrogen at baseline (47.73 ± 29.0 vs. 33.78 ± 13.04 mg/dL *p* < 0.001), blood urea nitrogen at 48 h (53.61 ± 34.11 vs. 32.71 ± 14.69 mg/dL *p* < 0.001). Also, neutrophils, lymphocytes, LDH, PCR and procalcitonin were significantly associated with the composite endpoint (Supplementary material Table 4^4^. Interestingly, age, AKI and comorbidities were not significantly associated with composite endpoint. Also AKI stages did not result in a significant increase in risk of COVID-19 disease severity. Conversely, drugs administrated for COVID-19 disease (oseltamivir, ritonavir/darunavir, hydroxychloroquine, and tocilizumab) were associated with composite endpoint, just as dexamethasone (OR 3.52 95% IC 1.84–6.71), but not for heparin (Supplementary material Table 5).

The effect of age on mortality (OR 9.71 95% IC 3.24–7.83) but not on composite endpoint was also confirmed after dichotomization of the variable (under and over 70 years). Analyzing all the individual components of the endpoint that express disease severity we found an inverse correlation for ETI (OR 0.34 95% IC 0.12–0.92), and CPAP (OR 0.37 95% IC 0.18–0.78) and a positive, but not significant, correlation FiO_2_ ≥ 60%. (see Table 3).

**Table 3:** Occurrence and comparison of endpoints in patients over and under 70 years old.

		Age < 70 years		Age > = 70 years		
		N	%	N	%	OR (95%CI)*
Inhospital mortality (0/1)	No	79	95.2	59	67.0	9.71 (3.24–29.1)
	Yes	4	4.8	29	33.0	
Oxygen flux FiO2 > 60%	No	61	73.5	53	60.2	1.83 (0.96–3.5)
	Yes	22	26.5	35	39.8	
ETI (0/1)	No	68	81.9	80	93.0	0.34 (0.12–0.92)
	Yes	15	18.1	6	7.0	
NPPV (0/1)	No	54	65.9	72	83.7	0.37 (0.18–0.78)
	Yes	28	34.1	14	16.3	
Composite endpoint	No	51	61.4	45	51.1	1.52 (0.83–2.80)
	Yes	32	38.6	43	48.9	

*Odds Ratio and 95% Confidence Interval. Reference category: absence of the condition or FiO2 ≤ 60.

ETI = endotracheal intubation, NPPV = non-invasive positive pressure ventilation.

### Predictive analysis

We identified the following most valuable variables to fit the predictive model and to achieve the best accuracy (%): age, sCr or eGFR (using different equations), blood urea nitrogen, comorbidities, leucocytes, neutrophils, and lymphocytes count, LDH, PCR, ferritin, procalcitonin, drugs. All the variables abovementioned were selected to predict the development of AKI, mortality, and/or severity of COVID-19 disease (composite endpoint).

The prediction performance of our cross-validated model is reported in terms of prediction accuracy (%) in Table 4.

**Table 4:** Predictive analysis.

	Accuracy (%)	Creatinine	CKD-EPI	BIS-1 over-70	BIS-1 over-80	MDRD
Overall population	Mortality	78.74	75.86	76.44	78.74	76.44
	Composite	59.77	66.09	64.37	60.92	66.09
	AKI	89.08	87.36	87.93	86.78	86.21
Age < 70 years	Mortality	88.37	90.70	-	-	91.86
	Composite	70.93	76.74	-	-	70.93
	AKI	89.53	94.19	-	-	91.86
Age ≥ 70 years	Mortality	69.32	67.05	67.05	61.36	71.59
	Composite	69.32	67.05	72.73	63.64	75.00
	AKI	81.82	82.95	72.73	77.27	84.09

We calculated the accuracy (%) of prediction for mortality, composite endpoint and AKI using only a single indicator of kidney functionality (serum creatinine or a specific eGFR) together with the other variables, in the overall population, in the population with age < 70 years, and in the population with age ≥ 70 years. The other predictors or variables are age, blood urea nitrogen, comorbidities, leucocytes, neutrophils, and lymphocytes count, LDH, PCR, ferritin, procalcitonin, drugs.

For the overall population and for the subset with age < 70 years, the accuracy reached higher values in predicting AKI than the other two binary outcomes (mortality, composite). On the other hand, the model gave a better prediction in the population with age < 70 years. For the patients < 70 years, the eGFR formulas enabled a good prediction of AKI and mortality with accuracies > 90%, and a slightly better performance to predict AKI making use of CKD-EPI, compared to the other eGFR methods (94.19% vs. 91.86%, respectively). Interestingly, in the population > 70 years, the accuracy of our model was globally low, and BIS-1 formulas showed a generally poorer performance to predict the outcomes of mortality and AKI, compared to CKD-EPI and MDRD. In addition, for the age > 70 years, the accuracy reached higher values in predicting AKI than the other two outcomes (mortality, composite).

For the overall population and for both age subsets (< 70 years, > 70 years), the preceding prediction results can suggest thatthe level of serum creatinine or the value of eGFR on admission could be considered goodpredictors of AKI onset during hospitalization.

## Discussion

In our study, we added new information and insights on the contribution of kidney dysfunction in COVID-19. We investigated and characterized several risk factors in COVID-19, with a specific focus on the relationship between age and kidney dysfunction on progression and severity of the disease. The most interesting results of our study, in a population of COVID-19 hospitalized patients, are the importance of AKI as independent risk factor for mortality, the identification of key variables for risk assessment of patients, and the evidence that the influence of baseline GFR on the clinical outcomes decreases with age, and it seems to not represent a major risk factor for elderly patients.

AKI occurred in one-third of hospitalized COVID-19 patients with a wide range among different studies, probably due to differences in study populations examined^12–15^. Based on the literature, incidence of AKI seems to be higher in COVID-19 patients compared to SARS-CoV2 negative patients^16,17^, confirming that it is not an epiphenomenon in severe or critical disease. CKD is a well-known risk factor for AKI and poor outcomes in different clinical settings and is frequently observed in the elderly that appear to be at higher risk of developing severe or critical COVID-19 disease as is observed in other patients affected by comorbidities associated with kidney dysfunction (diabetes, CVD, and hypertension)^18^. This is why it is challenging to discriminate between association and causality of kidney impairment and poor outcome in COVID-19.

Moreover, a precise risk assessment in COVID-19 patients is essential to allocate health resources during the pandemic emergency.

A recent study performed on the Danish population^5^, which differentiated between pre-existing CKD and AKI through national healthcare registries, showed an increasing risk for severe disease in CKD population. However, the risk for severe disease or death was low for early stages of CKD and when the variable “age” was included in the regressive model^5^. Uribarri et al. performed a registry study and they found that 30% of patients had kidney dysfunction on admission and this subgroup experienced a greater mortality rate compared with COVID-19 patients with normal kidney function^19^. An observational study showed that kidney impairment (eGFR < 60 mL/min) upon hospital admission was an independent risk factor for poor prognosis in COVID-19. Interestingly kidney impairment showed a greater impact in non-elderly compared to elderly patients^6^.

Others studies have reported a significant influence of age on the association between CKD and disease progression. The same influence was not found for mortality, but this may be due to a markedly younger median age, compared to our sample^20^.

Beyond COVID-19, various studies investigated the relationship between eGFR and clinical outcomes in the elderly, and the role of reduced GFR in this population is still debated among nephrologists ^9^. Some studies demonstrated that in elderly patients a GFR < 60 mL/min is not associated with increased risk for mortality or progression to end stage kidney disease (ESKD)^21,22^, while a meta-analysis found a U shape curve in the relationship between GFR and mortality for older age group (> 65 years) with a higher risk for GFR > 115 mL/min^23^.

Thus, a lower limit for CKD definition in the elderly has been proposed (e.g., eGFR < 45 mL/min)^16^, and different GFR equations have been developed and validated in this population. Recent studies evaluated the performance of different and more used equations for eGFR and found various pitfalls, limitations, and conflicting results in the association with short- and long-term hard outcomes^10,24,25^.

To investigate the correlation between age and kidney dysfunction, first we analysed age as a dichotomous variable, and we demonstrated that it influences mortality but not the composite endpoint, performed to evaluate the severity of the disease. However, analysing the single outcomes of the composite endpoint, we found a positive, but not significant, correlation with the need for high flux oxygen therapy (FiO_2_ > 60%; OR 1.83 95% IC 0.96–3.5). Conversely, we demonstrated an inverse correlation between age and C-PAP and age and EIT. This could partially be due to a minor eligibility of elderly patients to more invasive therapeutic approaches, and maybe to a reduced availability of medical resources during the pandemic outbreak. The non-randomized nature of this study, however, does allow us to clarify the reasons.

Age and AKI confirmed their important role as independent risk factors for COVID-19 mortality. In particular, patients experienced severe AKI showed eight-fold increased risk for mortality compared to non-AKI patients. Among this group two patients underwent continuous renal replacement therapy. In the relationship between age and mortality, eGFR acted as a possible confounder with an effect of diminishing the hazard risk of death in patients over 70 years.

We did not observe a significant impact of pharmacologic treatment on disease progression or mortality, with the exception of steroids. Indeed, all the drugs used were positively correlated with the severity of disease, probably because patients affected by pauci-symptomatic diseases were not referred to pharmacologic treatment. Steroid treatment, on the other hand, seemed to have a beneficial effect (OR 0.45 95% IC 0.19–1.07), consistent with previous reports^26^. We also analysed the role of Renin–angiotensin–aldosterone system inhibitors. In contrast with other studies we did not find any significant correlation with our outcomes, but a not significant increase in the risk of AKI with iACE (Supplementary material Tables 1, 3, 5)^27^.

We also developed a predictive analysis with a high accuracy for AKI and mortality in the subset of patients < 70 years. Interestingly, in patients ≥ 70 years the model showed a worse accuracy, and a worse accuracy of BIS-1 formula compared to CKD-EPI and MDRD. Although BIS-1 formula has been specifically developed in the elderly population, the diagnostic and predictive performance of this equation did not show significant superiority compared to CKD-EPI and/or MDRD^19^. In our population, MDRD showed the best predictive value, for AKI, severity of the disease and mortality, while BIS-1, which identifies almost twice the amount of patients with low GFR (< 60 mL/min)compared to the other formula (28.7% vs. 16.6%) showed the worst performance compared to MDRD and CKD-EPI.

The worse accuracy of our predictive analysis in patients ≥ 70 years could be due to a lack of variables of interest in the older population, such as albuminuria and BMI, and the reduced influence of our variables in the elderly, including GFR.

In conclusion, this study confirms the important role of AKI in COVID-19 progression. We believe that in COVID-19 hospitalized patients is mandatory to achieve an early detection of AKI, in order to identify patients at high risk for mortality and morbidity. Moreover, our study diminishes the role of kidney function impairment on admission in COVID-19 patients, especially in the elderly patients, where its contribution, compared with age and AKI, is of minor relevance.

Reasons behind this evidence are partially speculative. GFR estimation based on sCr levels is actually the most feasible method in the clinical routine, but it presents strong bias in specific clinical conditions. Indeed, low sCr levels could be measured in fragile and sarcopenic patients, thus in this specific population, GFR could be artifactually higher. Decreased GFR is a well-recognized negative prognostic factor for mortality and CVD morbidity, but malnutrition inflammation syndrome (MIS), in which creatinine generation is very low, is a strong CVD risk factor and correlates with mortality as well ^28^.

Moreover, as we discussed above, our population was characterized by a high percentage of elderly patients and a relatively high mean GFR value, which, in this setting, became prognostically relevant when under 45 mL/min. Prospective studies including patients with mild impairment of kidney function are need to strengthen this evidence.

Our study has several limitations: first of all, the retrospective nature of the study and the limited population size allow us to promote only new but exploratory insights regarding the relationship between kidney dysfunction and COVID-19 outcomes. Specific prospective studies are needed to strengthen these evidences. Pre-admission creatinine values and clinical history of CKD were not available thus, we were unable to determine CKD diagnosis. Moreover, we missed important variables such as albuminuria, proteinuria, BMI and fragile state index. In particular, nutritional status and body mass assessment is essential to further investigations into the relationship between kidney dysfunction and clinical outcomes in the older old affected by COVID-19.

Finally, the evaluation of the severity, through the composite endpoint, was affected by a bias of eligibility of older patients to invasive treatments, due to clinical decision making, and probably influenced by the availability of healthcare resources during the COVID-19 outbreak in Italy.

## Supplementary Information

Supplementary Information.

## Data Availability

The datasets generated and/or analysed during the current study are available from the corresponding author on reasonable request.

## References

[1] Zhou F (2020). Clinical course and risk factors for mortality of adult inpatients with COVID-19 in Wuhan, China: a retrospective cohort study. Lancet (London, England).

[2] Thakur B, Dubey P, Benitez J (2021). A systematic review and meta-analysis of geographic differences in comorbidities and associated severity and mortality among individuals with COVID-19. Sci Rep.

[3] Hardenberg JHB (2021). Critical illness and systemic inflammation are key risk factors of severe acute kidney injury in patients with COVID-19. Kidney Int. Reports.

[4] Liu, Y. F. *et al.* The chronic kidney disease and acute kidney injury involvement in COVID-19 pandemic: A systematic review and meta-analysis. *PLoS One* **16**, (2021). 10.1371/journal.pone.0244779 PMC7785235 33400721

[5] Carlson N (2021). Increased vulnerability to COVID-19 in chronic kidney disease. J. Intern. Med..

[6] Chen K (2021). Clinical outcomes of hospitalized COVID-19 patients with renal injury: a multi-hospital observational study from Wuhan. Sci Rep..

[7] Ozturk S (2021). Mortality analysis of COVID-19 infection in chronic kidney disease, haemodialysis and renal transplant patients compared with patients without kidney disease: A nationwide analysis from Turkey. Nephrol. Dial. Transplant..

[8] Ortiz A (2021). Chronic kidney disease is a key risk factor for severe COVID-19: A call to action by the ERA-edta. Nephrol. Dial. Transplant..

[9] Esposito C (2012). Loss of renal function in the elderly Italians: A physiologic or pathologic process?. J. Gerontol. Ser. A Biol. Sci. Med. Sci..

[10] Da Silva Selistre L (2019). Diagnostic performance of creatinine-based equations for estimating glomerular filtration rate in adults 65 years and older. JAMA Intern. Med..

[11] Levey AS (2009). A new equation to estimate glomerular filtration rate. Ann. Intern. Med..

[12] malERA Consultative Group on Basic Science and Enabling Technologies. A Research Agenda for Malaria Eradication: Basic Science and Enabling Technologies. *PLoS Med.* **8(1)**, e100039 (2011). 10.1371/journal.pmed.1000399 PMC3026698 21311584

[13] Cheng Y (2020). The incidence, risk factors, and prognosis of acute kidney injury in adult patients with coronavirus disease 2019. Clin. J. Am. Soc. Nephrol..

[14] Zheng X (2020). Prevalence of kidney injury and associations with critical illness and death in patients with COVID-19. Clin. J. Am. Soc. Nephrol..

[15] Neugarten J (2020). AKI in hospitalized patients with and without COVID-19: A comparison study. J. Am. Soc. Nephrol..

[16] Xu H (2021). Acute kidney injury and mortality risk in older adults with COVID-19. J. Nephrol..

[17] Moledina DG (2021). The association of COVID-19 with acute kidney injury independent of severity of illness: A multicenter cohort study. Am. J. Kidney Dis..

[18] Iaccarino, G. *et al.* Age and multimorbidity predict death among COVID-19 Patients: Results of the SARS-RAS study of the Italian society of hypertension. *Hypertension* **76**, (2020). 10.1161/HYPERTENSIONAHA.120.15324 32564693

[19] Uribarri A (2020). Impact of renal function on admission in COVID-19 patients: an analysis of the international HOPE COVID-19 (Health Outcome Predictive Evaluation for COVID 19) Registry. J. Nephrol..

[20] Wang B (2021). The involvement of chronic kidney disease and acute kidney injury in disease severity and mortality in patients with COVID-19: A meta-analysis. Kidney Blood Press. Res..

[21] Hallan SI (2012). Age and association of kidney measures with mortality and end-stage renal disease. JAMA J. Am. Med. Assoc..

[22] Delanaye P (2019). CKD: A call for an age-adapted definition. J. Am. Soc. Nephrol..

[23] Nitsch D (2013). Associations of estimated glomerular filtration rate and albuminuria with mortality and renal failure by sex: A meta-analysis. BMJ.

[24] Mandelli, S. *et al.* Mortality prediction in the oldest old with five different equations to estimate glomerular filtration rate: The Health and Anemia population-based study. *PLoS One* **10**, (2015). 10.1371/journal.pone.0136039 PMC4552830 26317988

[25] Torreggiani M (2021). Elderly patients in a large nephrology unit: Who are our old, old-old and oldest-old patients?. J. Clin. Med..

[26] Dexamethasone in hospitalized patients with Covid-19. *N. Engl. J. Med.* **384**, 693–704 (2021). 10.1056/NEJMoa2021436 PMC7383595 32678530

[27] Fabrizi F (2020). COVID-19 and acute kidney injury: A systematic review and meta-analysis. Pathogens..

[28] Jagadeswaran D (2019). Inflammation and nutritional status assessment by malnutrition inflammation score and its outcome in pre-dialysis chronic kidney disease patients. Clin. Nutr..

