# Supplementary material for: The role of kidney dysfunction in COVID-19 and the influence of age
Source: Sci Rep. 2022 May 23;12:8650. doi: 10.1038/s41598-022-12652-0 (PMC9125966; doi:10.1038/s41598-022-12652-0)
Supplement: Supplementary file 1 — Supplementary Information. [file 41598_2022_12652_MOESM1_ESM.docx]

**Supplementary materials-Table 1**. Qualitative variables associated to Acute Kidney Injury (AKI) in COVID-19 patients. Comparisons between patients with and without AKI.

|  | | **AKI** | | | |  |  |
| --- | --- | --- | --- | --- | --- | --- | --- |
|  |  | No (n=156) | | Yes (n=18) | |  |  |
|  |  | N | % | N | % | p | OR (95%CI) |
| **Gender** | F | 60 | 38.5 | 4 | 22.2 | 0.176 |  |
|  | M | 96 | 61.5 | 14 | 77.8 |  |  |
| **Comorbidity_Arterial hypertension (0/1)** | No | 86 | 55.1 | 3 | 16.7 | 0.002 | 6.14 (1.71-22.07) |
|  | Yes | 70 | 44.9 | 15 | 83.3 |  |  |
| **Comorbidity _Diabetes (0/1)** | No | 136 | 87.2 | 16 | 88.9 | 0.836 |  |
|  | Yes | 20 | 12.8 | 2 | 11.1 |  |  |
| **Comorbidity _COPD (0/1)** | No | 141 | 91.0 | 15 | 83.3 | 0.303 |  |
|  | Yes | 14 | 9.0 | 3 | 16.7 |  |  |
| **Comorbidity _CVD (0/1)** | No | 118 | 75.6 | 14 | 77.8 | 0.841 |  |
|  | Yes | 38 | 24.4 | 4 | 22.2 |  |  |
| **Comorbidity _Malignancies (0/1)** | No | 144 | 93.5 | 16 | 88.9 | 0.364 |  |
|  | Yes | 10 | 6.5 | 2 | 11.1 |  |  |
| **Comorbidity _Immunodeficiency_HIV (0/1)** | No | 150 | 96.8 | 18 | 100.0 | >0.90 |  |
|  | Yes | 5 | 3.2 | 0 | 0.0 |  |  |
| **Comorbidity _ Immunodeficiency _transplant (0/1)** | No | 154 | 99.4 | 18 | 100.0 | >0.90 |  |
|  | Yes | 1 | 0.6 | 0 | 0.0 |  |  |
| **ACEis (0/1)** | No | 135 | 87.7 | 13 | 72.2 | 0.074 | 2.73 (0.88-8.52) |
|  | Yes | 19 | 12.3 | 5 | 27.8 |  |  |
| **ARBs (0/1)** | No | 127 | 81.9 | 16 | 88.9 | 0.742 |  |
|  | Yes | 28 | 18.1 | 2 | 11.1 |  |  |
| **CCBs (0/1)** | No | 134 | 87.0 | 15 | 83.3 | 0.713 |  |
|  | Yes | 20 | 13.0 | 3 | 16.7 |  |  |
| **Nitrates (0/1)** | No | 151 | 98.1 | 18 | 100.0 | >0.90 |  |
|  | Yes | 3 | 1.9 | 0 | 0.0 |  |  |
| **CRRT (0/1)** | No | 153 | 100.0 | 16 | 88.9 | <0.001 | N.R. |
|  | Yes | 0 | 0.0 | 2 | 11.1 |  |  |
| **Oxygen flux FiO2>60%** | No | 106 | 67.9 | 9 | 50.0 | 0.128 |  |
|  | Yes | 50 | 32.1 | 9 | 50.0 |  |  |
| **NPPV (0/1)** | No | 113 | 73.9 | 14 | 77.8 | 0.719 |  |
|  | Yes | 40 | 26.1 | 4 | 22.2 |  |  |
| **CVC (0/1)** | No | 130 | 84.4 | 12 | 66.7 | 0.060 | 2.71 (0.93-7.91) |
|  | Yes | 24 | 15.6 | 6 | 33.3 |  |  |
| **ETI (0/1)** | No | 139 | 90.3 | 12 | 66.7 | 0.004 | 4.63 (1.52-14.13) |
|  | Yes | 15 | 9.7 | 6 | 33.3 |  |  |
| **Oseltamivir (0/1)** | No | 86 | 55.1 | 8 | 44.4 | 0.389 |  |
|  | Yes | 70 | 44.9 | 10 | 55.6 |  |  |
| **Ritonavir/Darunavir (0/1)** | No | 79 | 50.6 | 7 | 38.9 | 0.345 |  |
|  | Yes | 77 | 49.4 | 11 | 61.1 |  |  |
| **Hydroxychloroquine (0/1)** | No | 37 | 23.7 | 5 | 27.8 | 0.703 |  |
|  | Yes | 119 | 76.3 | 13 | 72.2 |  |  |
| **Antibiotics (0/1)** | No | 21 | 13.5 | 1 | 5.6 | 0.476 |  |
|  | Yes | 135 | 86.5 | 17 | 94.4 |  |  |
| **Tocilizumab (0/1/2)** | No | 136 | 87.7 | 17 | 100.0 | 0.222 |  |
|  | Yes | 19 | 12.3 | 0 | 0.0 |  |  |
| **Dexamethasone (0/1)** | No | 97 | 62.2 | 13 | 72.2 | 0.403 |  |
|  | Yes | 59 | 37.8 | 5 | 27.8 |  |  |
| **LMWH (0/1)** | No | 43 | 27.6 | 3 | 16.7 | 0.321 |  |
|  | Yes | 113 | 72.4 | 15 | 83.3 |  |  |

. OR are presented for statistically significant comparisons only. NR=Non Reliable estimate due to low numbers

COPD=Chronic obstructive pulmonary disease;; CVD=cardiovascular disease; ACEis= Angiotensin converting enzyme inhibitors; ARBs=Angiotensin converting enzyme inhibitors; CCBs= Calcium channel blockers; CRRT= continuous renal replacement therapy; CVC=central venous catheter; ETI=endotracheal intubation; LMWH=Low-molecular-weight heparin

**Supplementary materials-Table 2.** Quantitative variables associated to mortality. Comparisons between survivors and non survivors.

|  | **Mortality (0/1)** | | | |  |
| --- | --- | --- | --- | --- | --- |
|  | No (n=138) | | Yes (n=34) | | p* |
|  | Mean/ Median | SD/  IQR | Mean/ Median | SD/  IQR |  |
| **Age (years)** | 66.37 | 15.95 | 80.16 | 7.85 | <0.001 |
| **Creatinine (mg/dL)** | 0.89  0.83 | 0.39  0.26 | 1.14  1.11 | 0.41  0.45 | 0.009 |
| **Creatinine (micromol/L)** | 78.60  73.39 | 34.37  22.55 | 100.03  95.49 | 35.51  36.69 | 0.002 |
| **Creatinine 48 hours (mg/dL)** | 0.89  0.80 | 0.54  0.29 | 1.33  1.10 | 0.70  0.93 | 0.062 |
| **Creatinine 7 days (mg/dL)** | 0.86  0.79 | 0.34  0.31 | 1.49  1.25 | 0.98  0.79 | 0.003 |
| **eGFR (MDRD)** | 93.64 | 32.80 | 68.74 | 28.53 | <0.001 |
| **eGFR (CKD-EPI;BIS-1 over-80 years)** | 80.99 | 24.63 | 57.30 | 21.85 | <0.001 |
| **eGFR (CKD-EPI; BIS-1 over-70 years)** | 79.84 | 24.76 | 55.70 | 20.81 | <0.001 |
| **eGFR (CKD-EPI)** | 83.85 | 22.43 | 61.37 | 21.77 | <0.001 |
| **Blood urea nitrogen (mg/dL)** | 35.30 | 17.49 | 59.78 | 30.70 | <0.001 |
| **Blood urea nitrogen 48 hours (mg/dL)** | 36.29 | 19.54 | 67.86 | 39.47 | 0.002 |
| **White blood cells (n°/cc)** | 6842.8  5310 | 7265.0  3430 | 7867.6  7250 | 4815.1  2870 | 0.038 |
| **Neutrophiles (n°/cc)** | 4618.5  3550 | 3057.7  2900 | 6373.0  5060 | 4471.7  3120 | 0.018 |
| **Limphocytes (n°/cc)** | 1102.4 | 579.1 | 875.1 | 434.9 | 0.036 |
| **Platelets (n°)** | 214.82 | 87.05 | 204.91 | 104.04 | 0.573 |
| **LDH (IU/L)** | 264.99  242.5 | 97.98  129.50 | 338.80  287 | 171.81  183 | 0.071 |
| **GOT (IU/L)** | 36.58  29 | 26.93  22 | 55.52  45 | 49.08  31 | 0.008 |
| **GPT (IU/L)** | 37.15  27 | 35.16  27 | 41.29  28 | 47.60  29.5 | 0.741 |
| **CRP (mg/L)** | 60.52  39 | 64.69  77.39 | 109.75  113.2 | 82.07  79.9 | <0.001 |
| **Procalcitonine (ng/mL)** | .41  .10 | 1.35  .14 | 2.61  .20 | 7.94  .80 | 0.01 |
| **Ferritin (ng/mL)** | 717.38  499 | 785.34  564 | 1209.67  716.5 | 1175.65  2009 | 0.168 |
| **D-dimer (ng/mL)** | 1911.45  818 | 3870.72  1454 | 6108.08  1989.5 | 9079.59  5809 | 0.007 |
| **Systolic blood pressure (mmHg)** | 128.7 | 19.85 | 121.61 | 21.43 | 0.108 |
| **Diastolic blood pressure (mmHg)** | 75.20 | 11.71 | 68.50 | 13.12 | 0.012 |
| **Lenght of hospitalization (days)** | 26.39  21.5 | 20.52  21 | 20.29  13.5 | 19.24  15 | 0.030 |
| **Time between the onset of symptoms and discharge/death (days)** | 27.05 | 16.49 | 19.00 | 12.82 | 0.014 |

*Student’s t test

eGFR=Estimated Glomerular Filtration Rate; MDRD= Modification of Diet in Renal Disease Study; CKD-EPI=Chronic Kidney Disease Epidemiology Collaboration; BIS-1= Berlin Initiative Study 1 Equation; LDH=Lactate Dehydrogenase; GOT=Glutamic Oxaloacetic Transaminase; GPT= Glutamate Pyruvate Transaminase; CRP= C-Reactive Protein;

**Supplementary materials-Table 3.** Qualitative variables associated to mortality. Comparisons between survivors and non survivors.

|  | | **Mortality (0/1)** | | | |  |  |
| --- | --- | --- | --- | --- | --- | --- | --- |
|  |  | No (n=138) | | Yes (n=34) | |  |  |
|  |  | N | % | N | % | p* | OR (95%CI)** |
| **Gender** | F | 51 | 36.4 | 13 | 38.2 | 0.845 |  |
|  | M | 89 | 63.6 | 21 | 61.8 |  |  |
| **AKI** | No | 131 | 93.6 | 25 | 73.5 | 0.001 | 5.24 (1.89-14.5) |
|  | Yes | 9 | 6.4 | 9 | 26.5 |  |  |
| **Comorbidity_Arterial hypertension (0/1)** | No | 79 | 56.4 | 10 | 29.4 | 0.005 | 3.11 (1.38-6.99) |
|  | Yes | 61 | 43.6 | 24 | 70.6 |  |  |
| **Comorbidity _Diabetes (0/1)** | No | 127 | 90.7 | 25 | 73.5 | 0.007 | 3.52 (1.36-9.11) |
|  | Yes | 13 | 9.3 | 9 | 26.5 |  |  |
| **Comorbidity _COPD (0/1)** | No | 130 | 92.9 | 26 | 78.8 | 0.015 | 3.50 (1.22-10.04) |
|  | Yes | 10 | 7.1 | 7 | 21.2 |  |  |
| **Comorbidity _CVD (0/1)** | No | 115 | 82.1 | 17 | 50.0 | <0.001 | 4.60 (2.07-10.23) |
|  | Yes | 25 | 17.9 | 17 | 50.0 |  |  |
| **Comorbidity _Malignancies (0/1)** | No | 130 | 92.9 | 30 | 93.8 | >0.90 |  |
|  | Yes | 10 | 7.1 | 2 | 6.3 |  |  |
| **Comorbidity _Immunodeficiency_HIV (0/1)** | No | 135 | 96.4 | 33 | 100.0 | 0.585 |  |
|  | Yes | 5 | 3.6 | 0 | 0.0 |  |  |
| **Comorbidity _ Immunodeficiency _transplant (0/1)** | No | 139 | 99.3 | 33 | 100.0 | >0.90 |  |
|  | Yes | 1 | 0.7 | 0 | 0.0 |  |  |
| **ACEis (0/1)** | No | 120 | 85.7 | 28 | 87.5 | 0.793 |  |
|  | Yes | 20 | 14.3 | 4 | 12.5 |  |  |
| **ARBs (0/1)** | No | 118 | 84.3 | 25 | 75.8 | 0.244 |  |
|  | Yes | 22 | 15.7 | 8 | 24.2 |  |  |
| **CCBs (0/1)** | No | 119 | 85.0 | 30 | 93.8 | 0.189 |  |
|  | Yes | 21 | 15.0 | 2 | 6.3 |  |  |
| **Nitrates (0/1)** | No | 138 | 98.6 | 31 | 96.9 | 0.463^(1)^ |  |
|  | Yes | 2 | 1.4 | 1 | 3.1 |  |  |
| **CRRT (0/1)** | No | 137 | 99.3 | 32 | 97.0 | 0.350 |  |
|  | Yes | 1 | 0.7 | 1 | 3.0 |  |  |
| **Oxygen flux FiO2>60%** | No | 107 | 76.4 | 8 | 23.5 | <0.001 | 10.54 (4.36-25.49) |
|  | Yes | 33 | 23.6 | 26 | 76.5 |  |  |
| **NPPV (0/1)** | No | 105 | 76.1 | 22 | 66.7 | 0.266 |  |
|  | Yes | 33 | 23.9 | 11 | 33.3 |  |  |
| **CVC (0/1)** | No | 121 | 87.1 | 21 | 63.6 | 0.001 | 3.84 (1.62-9.12) |
|  | Yes | 18 | 12.9 | 12 | 36.4 |  |  |
| **ETI (0/1)** | No | 127 | 91.4 | 24 | 72.7 | 0.003 | 3.97 (1.51-10.45) |
|  | Yes | 12 | 8.6 | 9 | 27.3 |  |  |
| **Oseltamivir (0/1)** | No | 84 | 60.0 | 10 | 29.4 | 0.001 | 3.60 (1.60-8.10) |
|  | Yes | 56 | 40.0 | 24 | 70.6 |  |  |
| **Ritonavir/Darunavir (0/1)** | No | 77 | 55.0 | 9 | 26.5 | 0.003 | 3.39 (1.48-7.98) |
|  | Yes | 63 | 45.0 | 25 | 73.5 |  |  |
| **Hydroxychloroquine (0/1)** | No | 35 | 25.0 | 7 | 20.6 | 0.590 |  |
|  | Yes | 105 | 75.0 | 27 | 79.4 |  |  |
| **Antibiotics (0/1)** | No | 21 | 15.0 | 1 | 2.9 | 0.081^(1)^ |  |
|  | Yes | 119 | 85.0 | 33 | 97.1 |  |  |
| **Tocilizumab (0/1/2)** | No | 121 | 87.7 | 32 | 94.1 | 0.372^(1)^ |  |
|  | Yes | 17 | 12.3 | 2 | 5.9 |  |  |
| **Dexamethasone (0/1)** | No | 84 | 60.0 | 26 | 76.5 | 0.074 |  |
|  | Yes | 56 | 40.0 | 8 | 23.5 |  |  |
| **LMWH (0/1)** | No | 40 | 28.6 | 6 | 17.6 | 0.195 |  |
|  | Yes | 100 | 71.4 | 28 | 82.4 |  |  |

*Chi-square test or ^(1)^ Fisher exact test, as appropriate. ***Odds Ratio (OR) and 95% Confidence Interval. OR are presented for statistically significant comparisons only.

COPD=Chronic obstructive pulmonary disease; LMWH=Low-molecular-weight heparin; CVD=cardiovascular disease; ACEis= Angiotensin converting enzyme inhibitors; ARBs=Angiotensin converting enzyme inhibitors; CCBs= Calcium channel blockers; CRRT= continuous renal replacement therapy; CVC=central venous catheter; ETI=endotracheal intubation

**Supplementary materials-Table 4.** Quantitative variables associated to COVID-19 severity. Comparisons between patients with and without composite endpoint.

|  | **Composite endpoint** | | | |  |
| --- | --- | --- | --- | --- | --- |
|  | No (n=96) | | Yes (n=78) | | p* |
|  | Mean/ Median | SD/  IQR | Mean/ Median | SD/  IQR |  |
| **Age (years)** | 67.48 | 16.50 | 71.01 | 14.51 | 0.141 |
| **Creatinine (mg/dL)** | 0.85  0.81 | 0.25  0.25 | 1.05  .90 | 0.52  0.36 | 0.012 |
| **Creatinine (micromol/L)** | 74.78  71.62 | 22.23  22.11 | 92.96  79.58 | 45.69  30.06 | 0.004 |
| **Creatinine 48 hours (mg/dL)** | 0.83  0.80 | 0.28  0.23 | 1.14  0.89 | 0.80  0.54 | 0.034 |
| **Creatinine 7 days (mg/dL)** | 0.88  0.80 | 0.36  0.30 | 1.06  0.86 | 0.71  0.57 | 0.272 |
| **eGFR (MDRD)** | 96.65 | 34.04 | 78.80 | 29.91 | 0.001 |
| **eGFR (CKD-EPI;BIS-1 over-80 years)** | 81.69 | 24.67 | 70.04 | 25.86 | 0.004 |
| **eGFR (CKD-EPI; BIS-1 over-70 years)** | 80.26 | 24.94 | 69.07 | 25.65 | 0.005 |
| **eGFR (CKD-EPI)** | 85.33 | 21.68 | 72.37 | 24.80 | <0.001 |
| **Blood urea nitrogen (mg/dL)** | 33.78 | 13.04 | 47.73 | 29.00 | <0.001 |
| **Blood urea nitrogen 48 hours (mg/dL)** | 32.71 | 14.69 | 53.61 | 34.11 | <0.001 |
| **White blood cells (n°/cc)** | 7056.8  5150 | 8458.4  3545 | 6961.8  6015 | 3951.4  3610 | 0.0046 |
| **Neutrophiles (n°/cc)** | 4535.1  3380 | 3093.4  2800 | 5484.6  4400 | 3768.7  3500 | 0.017 |
| **Limphocytes (n°/cc)** | 1156.7 | 610.4 | 937.9 | 468.0 | 0.01 |
| **Platelets (n°)** | 218.56 | 88.34 | 205.84 | 92.86 | 0.362 |
| **LDH (IU/L)** | 237.73  222 | 71.85  89 | 330.03  300.5 | 141.30  169 | 0.001 |
| **GOT (IU/L)** | 33.60  29 | 20.92  21 | 48.26  36 | 42.15  26 | 0.008 |
| **GPT (IU/L)** | 34.13  26 | 29.34  26 | 42.85  28.5 | 45.94  30 | 0.193 |
| **CRP (mg/L)** | 49.82  27 | 54.10  65.20 | 94.81  68 | 80.83  99 | 0.001^(1)^ |
| **Procalcitonin (ng/mL)** | .19  .10 | .52  .08 | 1.51  .10 | 5.35  .34 | 0.061 |
| **Ferritin (ng/mL)** | 539.3  466 | 416.7  564 | 1076.7  643.5 | 1117.4  1223 | 0.096 |
| **D-dimer (ng/mL)** | 1421  770 | 1833  1168 | 3446  1182 | 6613  1814 | 0.098 |
| **Systolic blood pressure (mmHg)** | 126.64 | 20.09 | 127.38 | 20.79 | 0.843 |
| **Diastolic blood pressure (mmHg)** | 74.07 | 10.78 | 73.15 | 13.75 | 0.689 |
| **Lenght of hospitalization (days)** | 23  15 | 21  19 | 28  23 | 19  22 | 0.007 |
| **Time between the onset of symptoms and discharge/death (days)** | 22.44 | 15.73 | 29.16 | 15.93 | 0.01 |

*Student’s t test or ^(1)^Mann-Whitney test, as appropriate

eGFR=Estimated Glomerular Filtration Rate; MDRD= Modification of Diet in Renal Disease Study; CKD-EPI=Chronic Kidney Disease Epidemiology Collaboration; BIS-1= Berlin Initiative Study 1 Equation; LDH=Lactate Dehydrogenase; GOT=Glutamic Oxaloacetic Transaminase; GPT= Glutamate Pyruvate Transaminase; CRP= C-Reactive Protein;

**Supplementary materials-Table 5.** Qualitative variables associated to COVID-19 severity. Comparisons between patients with and without composite endpoint.

|  | | **Composite endpoint** | | | |  |  |
| --- | --- | --- | --- | --- | --- | --- | --- |
|  |  | No (n=96) | | Yes (n=78) | |  |  |
|  |  | N | % | N | % | p* | OR (95%CI)** |
| **Gender** | F | 33 | 34.4 | 31 | 39.7 | 0.465 |  |
|  | M | 63 | 65.6 | 47 | 60.3 |  |  |
| **AKI** | No | 88 | 91.7 | 68 | 87.2 | 0.334 |  |
|  | Yes | 8 | 8.3 | 10 | 12.8 |  |  |
| **Comorbidity_Arterial hypertension (0/1)** | No | 55 | 57.3 | 34 | 43.6 | 0.072 | 1.74 (0.95-3.17) |
|  | Yes | 41 | 42.7 | 44 | 56.4 |  |  |
| **Comorbidity _Diabetes (0/1)** | No | 86 | 89.6 | 66 | 84.6 | 0.327 |  |
|  | Yes | 10 | 10.4 | 12 | 15.4 |  |  |
| **Comorbidity _COPD (0/1)** | No | 88 | 91.7 | 68 | 88.3 | 0.461 |  |
|  | Yes | 8 | 8.3 | 9 | 11.7 |  |  |
| **Comorbidity _CVD (0/1)** | No | 76 | 79.2 | 56 | 71.8 | 0.258 |  |
|  | Yes | 20 | 20.8 | 22 | 28.2 |  |  |
| **Comorbidity _Malignancies (0/1)** | No | 88 | 91.7 | 72 | 94.7 | 0.433 |  |
|  | Yes | 8 | 8.3 | 4 | 5.3 |  |  |
| **Comorbidity _Immunodeficiency_HIV (0/1)** | No | 91 | 94.8 | 77 | 100.0 | 0.042 | N.R. |
|  | Yes | 5 | 5.2 | 0 | 0.0 |  |  |
| **Comorbidity _ Immunodeficiency _transplant (0/1)** | No | 95 | 99.0 | 77 | 100.0 | **>0.90** ^(1)^ |  |
|  | Yes | 1 | 1.0 | 0 | 0.0 |  |  |
| **ACEis (0/1)** | No | 84 | 87.5 | 64 | 84.2 | 0.536 |  |
|  | Yes | 12 | 12.5 | 12 | 15.8 |  |  |
| **ARBs (0/1)** | No | 82 | 85.4 | 61 | 79.2 | 0.285 |  |
|  | Yes | 14 | 14.6 | 16 | 20.8 |  |  |
| **CCBs (0/1)** | No | 84 | 87.5 | 65 | 85.5 | 0.706 |  |
|  | Yes | 12 | 12.5 | 11 | 14.5 |  |  |
| **Nitrates (0/1)** | No | 95 | 99.0 | 74 | 97.4 | 0.584^(1)^ |  |
|  | Yes | 1 | 1.0 | 2 | 2.6 |  |  |
| **CRRT (0/1)** | No | 94 | 100.0 | 75 | 97.4 | 0.201^(1)^ |  |
|  | Yes | 0 | 0.0 | 2 | 2.6 |  |  |
| **CVC** | No | 93 | 97.9 | 49 | 63.6 | <0.001 | N.R. |
|  | Yes | 2 | 2.1 | 28 | 36.4 |  |  |
| **Oseltamivir (0/1)** | No | 60 | 62.5 | 34 | 43.6 | 0.013 | 2.16 (1.17-3.97) |
|  | Yes | 36 | 37.5 | 44 | 56.4 |  |  |
| **Ritonavir/Darunavir (0/1)** | No | 59 | 61.5 | 27 | 34.6 | <0.001 | 3.01 (1.62-5.61) |
|  | Yes | 37 | 38.5 | 51 | 65.4 |  |  |
| **Hydroxychloroquine (0/1)** | No | 31 | 32.3 | 11 | 14.1 | 0.005 | 2.90 (1.35-6.26) |
|  | Yes | 65 | 67.7 | 67 | 85.9 |  |  |
| **Antibiotics (0/1)** | No | 21 | 21.9 | 1 | 1.3 | <0.001 | N.R. |
|  | Yes | 75 | 78.1 | 77 | 98.7 |  |  |
| **Tocilizumab (0/1/2)** | No | 90 | 95.7 | 63 | 80.8 | 0.002 | 5.36 (1.70-16.9) |
|  | Yes | 4 | 4.3 | 15 | 19.2 |  |  |
| **Dexamethasone (0/1)** | No | 73 | 76.0 | 37 | 47.4 | <0.001 | 3.52 (1.84-6.71) |
|  | Yes | 23 | 24.0 | 41 | 52.6 |  |  |
| **LMWH (0/1)** | No | 39 | 40.6 | 7 | 9.0 | <0.001 | 6.94 (2.89-16.7) |
|  | Yes | 57 | 59.4 | 71 | 91.0 |  |  |

*Chi-square test or ^(1)^Fisher exact test, as appropriate. ***Odds Ratio (OR) and 95% Confidence Interval. OR are presented for statistically significant comparisons only. NR=Non Reliable estimate due to low numbers

COPD=Chronic obstructive pulmonary disease; =cardiovascular disease; ACEis= Angiotensin converting enzyme inhibitors; ARBs=Angiotensin converting enzyme inhibitors; CCBs= Calcium channel blockers; CRRT= continuous renal replacement therapy; CVC=central venous catheter; ETI=endotracheal intubation; LMWH=Low-molecular-weight heparin; CVD
